# Effects of Growth Hormone and Insulin-Like Growth Factor-1 on Postoperative Muscle and Substrate Metabolism

**DOI:** 10.1155/2010/647929

**Published:** 2009-11-22

**Authors:** Folke Hammarqvist, Ingmar Wennström, Jan Wernerman

**Affiliations:** ^1^Gastrocentrum, Karolinska University Hospital Huddinge, K53, S-141 86 Stockholm, Sweden; ^2^CLINTEC (Institute of Clinical Science Intervention and Technology), The Karolinska Institutet, S-141 86 Stockholm, Sweden; ^3^Scandinavian Venous Centre, S-113 32 Stockholm, Sweden; ^4^Department of Anaesthesia and Intensive Care, Karolinska University Hospital Huddinge, B-32, S-141 86 Stockholm, Sweden

## Abstract

This study explored if a combined supplementation of GH and IGF-1 had an additive effect on whole body nitrogen economy, energy, substrate and skeletal muscle metabolism following surgical trauma. Patients were randomized to controls (C; *n* = 10), to GH (0.15 IU/kg/injection) (GH; *n* = 7) or GH combined with IGF-1 (40 *μ*g/kg/injection) subcutaneously twice a day (GH-IGF-1; *n* = 9) together with standardized parenteral nutrition. Muscle amino acids, glutathione and the ribosomal pattern reflecting protein synthesis, and nitrogen balance were measured. GH- and GH-IGF-1 groups showed lower urea and higher plasma glucose concentrations. Energy expenditure increased in the GH-group. GH-IGF-1 prevented a decrease in muscle polyribosomes indicating a preserved muscle protein synthesis. In the GH group unaltered BCAA and AAA levels were seen in muscle indicating an unchanged protein breakdown, while the other groups showed increased muscle concentrations postoperatively. Without statistically difference GH marginally improved the nitrogen balance, in terms of higher values, and growth factors improved the nitrogen balance when the shift in urea was taken into account. To conclude, growth factors influences urea metabolism, protein degradation and protein synthesis. There was no clearcut additional effect when combining GH and IGF-1 but the study was probably underpowered to outrule this and effects on nitrogen balance.

## 1. Introduction

Loss of lean body mass, and muscle tissue in particular, may in critically ill patients be detrimental, and it increases complications and impairs the clinical outcome. Therefore, nutritional therapies together with therapies reducing the catabolic burden and improving lean body mass are clinically important [1]. Growth hormone has major effects on metabolism and affects the utilization of substrates and changes the tissue specific metabolism [2]. Several earlier studies have shown its potential benefits with regard to protein metabolism, which also is reflected in clinically positive effects following surgery and in burn injuries [3–9]. However, a large multicenter study involving critically ill patients revealed an increased mortality in the GH-treated group indicating that its effects in situations with uncontrolled ongoing inflammation are associated with major negative consequences [10]. The present study was planned and completed before the result from the multinational study was reported. 

 Parts of the action of growth hormone are mediated through the insulin-like growth factor-1 (IGF-1). Administration of growth hormone induces a rise in circulating IGF-1 [11] that has important metabolic effects in stimulating glucose and amino acid uptake in muscle and improving muscle protein synthesis [12, 13]. In catabolic situations the levels of IGF-1 decrease while its binding proteins increases leading to a lower local IGF-1 activity, contributing to the decreased insulin sensitivity seen in catabolism [14–16]. 

 The metabolic effects of GH are at least in part mediated through the action of IGF-1 produced in the liver and in the peripheral tissues by the influence of growth hormone [17–19]. In skeletal muscle a reduced gene-expression of the GH-receptor is seen following surgical trauma [20]. This reduces the local IGF-1 synthesis, an effect that may be counteracted by GH supplementation [21]. The interrelation between GH, IGF-1 and IGF-binding proteins (GH/IGF-1/IGFBP-axis) is thus affected mirrored by a lower GH sensitivity, leading to lower IGF-1 and higher IGFBP levels that have been described in ICU patients leading to a lower IGF-1 bioavailability [14, 22]. In septic patients decreased production of IGF-1 has also been described [23]. The change in the GH/IGF-1/IGFBP-axis is possible to counteract by amino acid supplementation [24]. A similar effect on the GH/IGF-1/IGFBP-axis is seen in cardiac surgery when a glucose-insulin-potassium (GIK) infusion is given peroperatively [25]. IGF-1 levels have also been shown to be possible to increase in catabolic patients, either by GH administration or by giving IGF-1 [11, 26]. 

 A combination of growth hormone and IGF-1 has a theoretical beneficial potential since the decreased insulin sensitivity induced by growth hormone may be outbalanced by an addition of IGF-1. GH increases the binding protein for IGF and concomitant administration may therefore increase the bioavailability of IGF-1 and increase its effects on the peripheral tissues. Combination of growth hormone and IGF-1 with glutamine containing TPN has been shown to improve protein balance in critically ill patients [27, 28], but the combined effect on nitrogen, amino acid, and protein metabolism has so far not been explored following surgical trauma. This study was designed to elucidate the effects on muscle amino acid, glutathione, and protein metabolism of growth hormone alone or in combination with IGF-1 given together with continuous nutritional supplementation following colonic resection as a human model of trauma. 

 The present study was discontinued when the report of an increased mortality of ICU patients given GH was published [10], but since the results may be of general interest we hereby communicate this almost 10 years later.

## 2. Patients and Methods

 As a model of elective trauma metabolically healthy individuals under the age of 80, undergoing elective colonic resection because of nonspread colonic cancer or earlier diverticulitis, were included in this randomized and blinded study (*n* = 26). Colonic resection was chosen as a surgical trauma model leading to muscle catabolism, used in earlier studies on muscle and nitrogen metabolism [29, 30]. The operation is standardized, and metabolic changes indicating a catabolic situation are seen during the early postoperatve period [29, 30]. The characteristics of the patients and of the operative procedures are shown in Table 1. They were preoperatively weight stable, not under medication affecting the metabolism, and none of them had signs of ongoing inflammation. The patients were postoperatively given isocaloric (28 kcal/kg/24 h) and isonitrogenous (0.15 g N/kg/24 h) TPN (total parenteral nutrition), not containing glutamine, for 3 days as a continuous infusion. One group served as a control group (*n* = 10), another group was given GH postoperatively (GH; *n* = 7) twice a day subcutaneously (0.15 IU/kg body weight/injection) and a third group (GH-IGF-1; *n* = 9) was given the same amount of GH and IGF-1 (40 *μ*g/kg body weight/injection) twice a day. This dosages has in earlier studies been shown to influence protein and substrate metabolism [5, 11, 31–35]. The study was designed to include 10 patients in each group, but when the use of growth hormone in catabolic patients was stopped by the results from the multicenter study on ICU patients [10] inclusion of patients in the present study was stopped.

Preoperatively, after induction of anaesthesia, a muscle biopsy was taken with percutaneous technique from the vastus lateralis femoris muscle. The muscle tissue of about 200 mg wet weight was divided into portions for the analyses of the amino acid and glutathione concentration and the content and size distribution of ribosomes, reflecting protein synthesis. All visible fat and connective tissue were removed from the tissue sample and the muscle tissue was weighed on an electrobalance and then plunged into liquid nitrogen and thereafter stored at −80°C, until analysis. A second biopsy was taken on the third postoperative day in local anaesthesia confined to the skin and fascia only. Daily blood samples were taken for determination of the concentrations of amino acids. Urine was collected in 24 hour portions for the determination of the cumulated nitrogen losses. The study protocol has been approved by the Swedish Medical Products Agency (Läkemedelsverket) and by the Ethical Committee of the Karolinska Institute, Stockholm, Sweden. The patients were informed about the study procedure and of the possible risks involved before consent was obtained.

### 2.1. Indirect Calorimetry

Energy expenditure and the respiratory quotient were measured by indirect calorimetry preoperatively and on the second postoperative day (Deltatrac, Datex Oi, Helsinki, Finland). The first measurement was made after an overnight fast before premedication. The second measurement was made during continuous TPN treatment on the second postoperative day. The patients were measured during a 30-minute long period after 30 minutes of bedrest. Although the substrate availability may be different comparing these two situations the patients were measured in a standardized way that allows comparisons between the three groups.

### 2.2. Glucose, Urea, and CRP

Daily samples for determination of glucose, urea, and CRP were taken. Glucose was determined by an enzymatic colorimetric method, urea by a kinetic absorbance method and CRP by a quantitative immunological method. All analyses were performed using routine analyses at the hospital.

### 2.3. Nitrogen Balance

The whole-body nitrogen balance was determined by subtracting the measured nitrogen excretion in urine every 24-hour from the amino acid nitrogen content in the TPN. The extrarenal losses were approximated to be 1.5 g/24 hours that was included in the calculations. 

 The urine was acidified and refrigerated immediately after voiding. After collecting the 24-hour portions the volume was measured and aliquots were stored at −20°C prior to analysis of the daily losses of urea (Urease Enzymatic's UV test, Boeringer, Mannheim, Germany) ammonia (GLD, kinetic UV test Merk, Darmstadt, Germany), and creatinine (Jaffé reaction, Beckman ASTRA, Palo Alto, USA). The total nitrogen excretion was determined using chemoluminescence (771 C Pyroreactor, 720 C Nitrogen Detector; ANTEK, Houston, Texas, USA). 

 Since treatment of growth factors significantly decreased the urea levels postoperatively, the contribution of the whole body urea shift was also taken into account expressing a urea related cumulated nitrogen balance [36]. Changes both in total body water and urea were taken into account in calculating the Urea-related nitrogen balance. In order to assess total body water Body Impedance Analysis (BIA) was used. The Urea related Nitrogen balance was calculated as follows:

- Urea related N balance = N  intake − N  urine − N  extrarenal  (approximated  to  be  1.5 g N/24 h) − Diff  urea.
- Diff urea = (urea end −  urea start) × total body water (start) × 0.028 + (body water end–body water (start)) × urea end × 0.028. (urea is expressed as mmol/L).
- Extrarenal nitrogenlosses (N extrarenal) are estimated to be 1.5 grams per day.

### 2.4. Amino Acids

Muscle tissue with a weight between 20–40 mg was used for analysis. The frozen muscle biopsy specimen was homogenized in 4% sulphosalicylic acid (SSA), containing norleucine as internal standard. The procedure is described in details elsewhere [37]. The amino acids were separated on an Ultropae 8 Lithium form ion exchange resin (Biochrom) using lithium citrate buffers. The amino acids were detected and quantified by postcolumn derivatization with o-phthaldialdehyde (OPA) and fluorescent detection at ex 350 nm em 420 nm. The concentrations of the amino acids were expressed as mmol/kg wet weight in muscle.

### 2.5. Glutathione and Thiols

Muscle tissue with a weight between 20 and 40 mg was used for analysis. Sample preparation and derivatization have been described in details elsewhere [38]. The frozen muscle tissue was homogenized and deproteinized in 6.5% sulphosalicylic acid (SSA) in a glass homogenizer on ice. The homogenate was centrifuged at 3.000× g for 15 minutes at 4°C. The protein precipitate was later used for the determination of protein-bound GSH and CySH. Samples of GSH and CySH standards or SSA-soluble fraction from muscle biopsies (100 *μ* *L*) were mixed with monobrombimane (mBBr) (8 *μ*M in sodium *N*-ethylmorpholine pH 8.0, 100 *μ* *L*), allowed to react for 5 minutes in the dark. Thereafter the reaction was stopped by the addition of 100% SSA (10 *μ* *L*). Total glutathione (GSH + GSSG), total cysteine and precipitated protein were also evaluated by the present method by performing a reduction step of GSSG and CySS with dithiothreitol (DTT) after protein precipitation. 

 The HPLC separation of thiol-bimane adducts was achieved on a column (150 × 4.5 mm) packed with 3 *μ*m octadodecylsilica reversed-phase resin, followed by fluorescent detection at ex 394 nm, em 480 nm.

### 2.6. Protein Synthesis

The protein synthesis in skeletal muscle tissue was studied by the ribosome method [39]. Muscle tissue specimens of 50–60 mg wet weight were used. The samples were homogenized in a medium containing a ribonuclease inhibitor and then centrifuged for 10 minutes at 1.500 g. The pellet was saved for DNA determination by a fluorescence method. The supernatant was ultracentrifuged for 2 hours at 102.000 g. The pellet containing the ribosomal particles was resuspended in 200 *μ* *L* of a medium containing a ribonuclease inhibitor. The ribosome concentration was determined spectrophotometrically at 260 nm and was expressed as optical density (OD) units per milligram of DNA. The remainder of the ribosomal suspension, 150 *μ* *L*, was layered onto a gradient of sucrose between 0.4 M and 1.5 M. The tubes were ultracentrifuged for 60 minutes in a swing-out rotor. Afterwards the gradient was displaced through a continuous-flow cuvette and the absorbance at 260 nm was recorded to show the distribution of ribosomes and polyribosomes in the sucrose density gradient. The area under the curve was measured and the percentage of polyribosomes in the total ribosome area was calculated.

### 2.7. Statistical Analysis

The values are expressed as medians with lower and upper quartiles. A nonparametric ANOVA (Kruskal-Wallis ANOVA) was used followed by Wilcoxon test for post hoc comparisons within the groups and Mann-Withney U-test for comparisons between the controls and the groups given growth factors. A *P*-value less than .05 was considered as significant. Borderline significance (*P* < .10) was also indicated and discussed. 

 This was a pilot study in which the effects on the primary effect variables were not known and therefore a power analysis was not perforned. Since the variation coefficient was <5% for the amino acid analyses, <10% for the ribosome analyses, and <8% for the analyses of glutathione, groups including 10 individuals each was considered sufficient to detect a difference of a larger magnitude as the coefficient of variation in paired samples.

## 3. Results

### 3.1. Energy Expenditure and Glucose (Tables 2 and 3)

The energy expenditure increased postoperatively in the GH-group by 15% (*P* = .024) but was unaltered in the other groups. No statistically significant changes were seen in respiratory quotient within or between the groups, reflecting that the relation between substrates used for oxidation was not influenced by the surgical trauma or administration of growth factors. 

Plasma glucose concentrations increased postoperatively in all group. The controls were normalized on the second postoperative day, while levels in the GH and IGF-1/GH groups remained elevated throughout the study period.

### 3.2. Urea and Nitrogen Balance (Tables 3 and 4)

Plasma urea decreased in both the GH- and GH-IGF-I groups on the postoperative day 2 by 43% and 39% and on day 3 by 50% and 54% , respectively, compared to the control group (*P* < .05) in which no change was seen. The cumulated postoperative nitrogen balance was positive in all groups without any differences between the groups. 

The urea corrected whole body cumulated nitrogen balance (expressed as g N) was numerically more positive in the GH group (11.43 (7.45–12.87)) compared to the control group (6.84 (3.19–8.96)) and compared to the GH-IGF-1 group ((8.92 (5.82–18.27)) but without reaching a significant level, *P* = .063 and *P* = .22, respectively.

### 3.3. Amino Acids (Tables 5 and 6)

Muscle glutamine decreased in the control group by 28 %, by 24% in the GH-group, and by 28% in the GH-IGF-1-group (*P* < .05). In parallel muscle BCAA increased by 44% in the control group, by 39% (*P* < .05) in the GH-IGF-1 group while no change was seen in the GH-group. A similar increase was also seen in the aromatic amino acids in muscle by 61% in the control group and by 46% (*P* < .05) in the GH-IGF-1 group. No change was seen in the GH-group. Plasma glutamine concentration decreased by 17% (*P* < .05) in the GH-IGF-1 group while the level was unchanged in the other groups. 

### 3.4. Muscle Glutathione (Table 7)

Total glutathione (tGSH) and reduced glutathione (GSH) decreased in the GH group by 16% and 12%, respectively, (*P* < .05) and by 15% and 19%, respectively, in the GH-IGF-1 group (*P* < .05). In the controls a numerical decrease by 13% was seen for tGSH, that did not attain statistical significance (*P* = .06) while GSH was unaltered. 

### 3.5. Ribosomes (Table 8)

The concentrations of total ribosomes did not change in any of the groups postoperatively.

The polyribosome concentrations decreased by 19 % in the control group and by 28 % in the GH group (*P* < .05) while the concentration was unchanged in the GH-IGF-1 group.

## 4. Discussion

In a human model of elective trauma the metabolic effects of GH either given alone or in combination with IGF-1, together with total parenteral nutrition during three days postoperatively were evaluated. Growth factors increased the energy expenditure, increased the glucose and lowered the urea levels. GH preserved the levels of BCAA and AAA in skeletal muscle indicating that protein breakdown was unaltered compared to increased postoperative levels in the other groups. A combination of GH and IGF-1 preserved the level of polyribosomes postoperatively indicating an unaltered protein synthesis compared to decreases levels in the other groups. 

 To combine growth hormone with either insulin or IGF-1 may be advantageous since GH increases peripheral insulin resistance and secondly since surgical trauma furthermore increases GH resistance with lower levels of circulating IGF-1. The effect of IGF-1 given alone in catabolic conditions is not as clear-cut as during growth hormone treatment. In some studies IGF-1 given alone postoperatively showed no effect on nitrogen [26, 36]. IGF-1 treatment during 5 days postoperatively reduced plasma insulin and glucose levels, indicating an improved insulin sensitivity following surgery [35]. 

 In the present study a daily supply of GH (0.3 IU/kg/day) and IGF-1 (80 *μ*g/kg/day) was chosen since these amounts have shown effects on protein and substrate metabolism in earlier studies during the postoperative period [5, 11, 31–35]. 

 Plasma glucose levels increased in both groups given growth factors as compared to the control group. This indicates an increased insulin resistance by growth factor treatment. A tendency, although not statistically significant, towards higher levels in the GH-group compared to the GH-IGF-1-group was observed. 

 A sufficient energy and protein supply is a prerequisite for an optimal IGF-I effect and response to growth hormone [40]. When compared to the resting energy consumption by indirect calorimetry an excess of energy in the range between 400 to 600 kcal per day was given in the present study every day. This amount covers the energy consumption related to the basal physical activity postoperatively. Treatment with growth factors increased the resting energy expenditure postoperatively compared to the control group without changing the RQ. 

 This may be explained by the action of growth factors, increasing the metabolism of substrates. Both growth hormone and IGF-I have in earlier studies been shown to increase the resting energy metabolism following surgery and in healthy volunteers [41–43]. Hyperglycemia has in mitochondrial studies been shown to affect mitochondrial metabolism and substrate and energy metabolism and increase the action of the uncoupling proteins [44]. This may also be a contributing factor to the increased energy metabolism seen in the groups given growth factors. 

 Urea levels decreased during the study period in both the GH- and GH-IGF-1 groups mirroring a lower urea-formation, an indirect sign of a decreased protein breakdown. Since the shift in whole body urea was significant in the GH- and GH-IGF-1 groups this was taken into account in a calculation determining the cumulated nitrogen balance related to the shift in urea. A tendency of an improved whole body nitrogen balance during the study period was seen in the groups given growth factors, the effect being close to significant in the GH-group. In all groups a positive nitrogen balance was seen which may be explained by continuous administered parenteral nutrition. This effect on nitrogen balance has earlier been reported when parenteral nutrition is administered continuously [45]. 

 In all three groups a similar decline in muscle glutamine was seen postoperatively. This decline in glutamine was not that extensive as has been shown in control groups in earlier glutamine studies receiving a similar amino acid composition as in the present study [30, 46]. There is no clear explanation to this, but it is plausible that the continuous administration of parenteral nutrition optimizes the metabolic conditions to an extent that reduces the changes in catabolic parameters. In the present study conventional TPN was given without glutamine since that was not a clinical routine at the time of the study. 

 A decline in muscle free glutamine has earlier been reported to be reduced by GH-treatment [5] but this finding was not reproduced. Changes in the total sum of BCAA, aromatic and essential amino acid concentrations differed between the groups. An increase in BCAA and AAA indicating an increased proteolysis was observed in the control group and in the group given GH together with IGF-1. The sum of essential amino acids in muscle increased in the control group. These changes indicate that protein degradation was reduced by GH treatment but not by the combined therapy. 

 The total amount of glutathione and its reduced form decreased postoperatively in all groups with the exception of maintained levels of reduced glutathione in the control group. No statistical differences were observed between the groups. This observation is in line with results from earlier studies in which muscle glutathione levels are affected by surgical trauma [30]. 

 Muscle protein synthesis was assessed by determination of the total amount and size distribution of ribosomes in muscle tissue. The concentration of polyribosomes reflects the protein synthesis activity in the tissue studied [32, 47]. The polyribosome concentrations decreased in the control and GH-group but were statistically unchanged in the GH-IGF-1 group. However using a nonparametric ANOVA no differences were seen between the three groups, making this observation not statistically robust enough. 

 There are historically studies with clinical beneficial effects with the use of GH [3, 6, 7, 9], but when used in a heterogeneous group of ICU patients with ongoing acute general inflammation an increased mortality is seen [10]. The mechanism behind that adverse effect may be increased insulin resistance, uncontrolled hyperglycemia, uncontrolled nutritional supply, a triggering of the inflammatory response, and a shortage of substrates for the immune system induced by a decreased peripheral release of glutamine. 

 It is important to consider a proper timing with regard to the inflammatory response, and a proper glucose control and to pay attention to the nutritional treatment. This has been elucidated in a recent study presenting metabolically positive effects when GH was administered in a pulsative way together with glutamine in ICU patients [48]. Despite the negative effect of GH treatment used in unselected ICU patients earlier there may still be a future for GH- treatment in selected ICU patients. 

 The present study showed effects on muscle amino acids indicating a lower protein degradation when GH was used, an prevention of a drop in postoperative protein synthesis when the combination of GH- and IGF-1 was given. Growth factors have a clear effect on plasma urea indicating reduced gluconeogenesis. Even when the change in urea was taken into consideration a significant difference was not seen between the control and groups given growth factors. The patients were all in positive nitrogen balance, a situation that was not further pronounced by treatment with growth factors.

## Figures and Tables

**Table 1:** Characteristics of the patients.

	Control	GH	GH-IGF
Age (years)	72 (70–74)	70 (47–76)	69 (66–73)
Gender male/female	4/6	4/3	5/4
BMI	23.4 (20.9–25.1)	24.7 (22.7–28.8)	26.4 (22.4–28.1)
Op time (min)	90 (80–105)	95 (65–120)	90 (75–100)
Bleeding (mL)	175 (150–300)	200 (150–500)	280 (200–300)
Days of hospital stay	6.5 (5.0–7.0)	5.0 (4.0–6.0)	7 (6-7)

**Table 2:** Respiratory quotient (RQ) and resting energy expenditure (REE) preoperatively and on the 2nd postoperative day. *P *< .10 are given as numbers indicating changes within the groups. Values are given as medians and quartiles.

		D0	D2
RQ	Control	0.88 (0.85–0.96)	0.91 (0.87–0.95)
	GH	0.89 (0.87–0.95)	0.87 (0.85–0.94)
	GH-IGF-1	0.88 (0.84–0.90)	0.89 (0.79–0.94)

REE	Control	1355 (1140–1450)	1380 (1270 –1650)
	GH	1520 (1200–1650)	1750 (1610–1970) *P* = .024
	GH-IGF-1	1470 (1240–1650)	1930 (1550–1970) *P* = .078
			

**Table 3:** Plasma glucose, urea and CRP levels pre- and postoperatively.

		D0	D1	D2	D3
Glucose (mmol/L)	Control	4.9 (4.3–5.4)	8.2 (6.2–9.8)*	6.6 (5.6–7.2)	5.8 (5.3–6.5)
	GH	4.6 (3.9–5.2)	8.2 (6.8–11.1)*	8.2 (6.7–10.9)*	9.1 (7.0–11.2)*#
	GH-IGF-1	4.8 (4.7–5.2)	8.7 (7.3–8.9)**	7.4 (6.5–9.9)**	7.4 (6.9–12.6)**#

Urea (mmol/L)	Control	4.0 (3.1–5.2)	4.4 (3.1–5.3)	3.4 (3.1–3.5)	3.4 (3.2–4.1)
	GH	4.0 (3.1–6.3)	3.1 (2.9–5.8)	2.3 (1.9–3.5)*	2.0 (1.4–2.9)*#
	GH-IGF-1	4.1 (3.5–4.9)	4.4 (3.5–4.9)	2.5 (2.0–2.9)*	1.9 (1.4–2.2)**#

CRP (mg/L)	Control	7 (5–13)	59 (55–91)**	104 (94–130)**	67 (59–109)**
	GH	5 (5–14)	46 (39–102)*	106 (43–145)*	69 (29–88)*
	GH-IGF-1	12 (5–20)	82 (66–86)**	135 (82–155)**	91 (53–114)**

*and **indicate a postoperative difference within the groups (*P* < .05 and *P* < .01) as compared to D0, and # indicates a difference between the controls and the groups given growth factors (*P* < .05). Values are given as medians and quartiles.

**Table 4:** Nitrogen balance during the postoperative period (g N). *P *< .10 are given as numbers indicating differences between the groups. Values are given as medians and quartiles.

	Control	GH	GH-IGF-1
N-balance day 1	2.84 (1.03–3.82)	4.14 (2.89–5.08)	3.04 (1.79–4.52)
N-balance day 2	0.44 (−0.80–1.84)	0.87 (-2.68–2.00)	1.25 (0.27–1.83)
N-balance day 3	2.04 (0.83–3.63)	3.43 (2.45–4.89)	3.09 (1.88–7.34)
Cumulated N-balance	6.28 (−0.01–8.26)	8.22 (5.34–9.74)	7.41 (3.39–14.11)
Cumulated N-bal urea	6.84 (3.19–8.96)	11.43 (7.45–12.87) *P* = .063	8.92 (5.82–18.27)

**Table 5:** Muscle amino acids (mmol/kg ww muscle). Glutamine (Gln), branched chain amino acids (BCAAs), aromatic amino acids (AAAs), essential amino acids (EAAs), total sum of amio acids (Tot AAs).

		D0	D3
Gln	Control	11.63 (10.46–12.87)	8.43 (8.14–9.07)**
	GH	11.49 (10.27–13.07)	8.75 (6.85–11.07)*
	GH-IGF-1	12.03 (10.72–12.93)	8.64 (10.99–6.57)**

BCAA	Control	0.38 (0.33–0.43)	0.55 (0.54–0.59)**
	GH	0.47 (0.38–0.51)	0.51 (0.44–0.55) #
	GH-IGF-1	0.36 (0.35–0.39)	0.50 (0.48–0.60)**

AAA	Control	0.13 (0.97–1.47)	0.21 (0.19–0.23)**
	GH	0.15 (0.13–0.16)	0.17 (0.16–0.20)
	GH-IGF-1	0.13 (0.10–0.15)	0.19 (0.17–0.20)*

EAA	Control	2.18 (2.07–2.43)	2.84 (2.53–3.12)**
	GH	2.16 (1.87–2.24)	2.26 (2.03–2.68) #
	GH-IGF-1	2.11 (1.85–2.57)	2.54 (2.28–2.72)

Tot AA	Control	21.26 (19.81–24.06)	18.69 (17.58–20.12)
	GH	21.60 (18.77–23.62)	19.69 (14.36–21.78)
	GH-IGF-1	21.84 (19.48–23.00)	19.53 (16.27–20.68)

*and **indicate a postoperative difference within the groups (*P *< .05 and *P * < .01) as compared to D0, and # indicates a difference between the controls and the groups given growth factors (*P *< . 05). Values are given as median and quartiles.

**Table 6:** Plasma amino acids (*μ*mol/L). Glutamine (Gln), branched chain amino acids (BCAAs), aromatic amino acids (AAAs), essential amino acids (EAAs), total sum of amio acids (Tot AAs).

		D0	D3
Gln	Control	554 (491–640)	510 (454–530)
	GH	565 (511–606)	554 (436–568)
	GH-IGF-1	581 (519–593)	478 (421–485)*

BCAA	Control	384 (258–417)	430 (376–473)
	GH	405 (359–422)	416 (323–436)
	GH-IGF-1	367 (280–393)	409 (362–447)

AAA	Control	102 (85–115)	146 (127–157)**
	GH	114 (90–122)	118 (109–133)#
	GH-IGF-1	109 (84–134)	121 (116–132)

EAA	Control	983 (694–1054)	1094 (1016–1157)
	GH	1010 (873–1126)	930 (806–1057)
	GH-IGF-1	932 (892–1061)	965 (912–1039)

Tot AA	Control	2123 (1720–2381)	2100 (2029–2389)
	GH	2160 (1990–2397)	2129 (1722–2406)
	GH-IGF-1	2254 (1991–2338)	2011 (1907–2113)

*and **indicate a postoperative difference within the groups (*P* < .05 and *P* < .01) as compared to D0, # and indicates a difference between the controls and the groups given growth factors (*P* < .05). Values are given as median and quartiles.

**Table 7:** Glutathione concentrations and redox-status of glutathione in skeletal muscle (mmol(kg ww muscle)). Total glutathione (tGSH), reduced glutathione (GSH), oxidized glutathione (GSSG), and the redox-status of glutathione (GSH/tGSH).

		D0	D3
tGSH	Control	2.02 (1.78–2.16)	1.75 (1.59–1.95) *P* = .06
	GH	2.03 (1.97–2.43)	1.71 (1.36–1.88)*
	GH-IGF-1	1.91 (1.71–1.98)	1.62 (1.40–1.79) *P* = .051

GSH	Control	1.67 (1.49–1.86)	1.52 (1.30–1.76)
	GH	1.65 (1.52–2.16)	1.45 (1.02–1.74)*
	GH-IGF-1	1.65 (1.50–1.73)	1.33 (1.25–1.47)*

GSSG	Control	0.17 (0.13–0.18)	0.13 (0.11–0.16)
	GH	0.18 (0.10–0.22)	0.15 (0.07–0.17)
	GH-IGF-1	0.13 (0.10–0.15)	0.15 (0.09–0.18)

GSH/tGSH	Control	0.83 (0.80–0.88)	0.86 (0.82–0.89)
	GH	0.83 (0.77–0.90)	0.86 (0.75–0.91)
	GH-IGF-1	0.85 (0.84–0.90)	0.83 (0.81–0.87)

*indicates a postoperative difference within the groups (*P* < .05 and *P* < .01). *P* < .10 are given as numbers. Values are given as median and quartiles.

**Table 8:** Ribosome and polyribosome concentrations (OD/mg DNA) in skeletal muscle.

		D0	D3
Tot rib	Control	39.4 (35.1–48.5)	35.4 (30.7–43.4)
	GH	39.3 (33.2–46.7)	36.7 (30.8–38.8)
	GH-IGF-1	38.0 (34.1–38.8)	36.8 (33.6–44.8)

Poly rib	Control	18.7 (14.6–21.6)	15.1 (12.8–19.7)*
	GH	20.9 (19.8–21.6)	15.0 (11.6–17.1)*
	GH-IGF-1	16.8 (16.3–18.2)	15.7 (14.9–20.5)

*indicates a postoperative difference within the groups (*P* < .05) as compared to D0. Values are given as median and quartiles.
